# Gypenosides ameliorate high-fat diet-induced nonalcoholic fatty liver disease in mice by regulating lipid metabolism

**DOI:** 10.7717/peerj.15225

**Published:** 2023-04-11

**Authors:** Tingting Zhou, Ligang Cao, Yimei Du, Lin Qin, Yanliu Lu, Qianru Zhang, Yuqi He, Daopeng Tan

**Affiliations:** 1 Guizhou Engineering Research Center of Industrial Key-technology for Dendrobium Nobile, Zunyi Medical University, Zunyi Medical University, Zunyi, Guizhou, China; 2 Joint International Research Laboratory of Ethnomedicine of Ministry of Education, Zunyi Medical University, Zunyi Medical University, Zunyi, Guizhou, China

**Keywords:** Gypenosides, NAFLD, Transcriptome, High-fat diet, Data mining

## Abstract

Gypenosides (GP), extracted from the traditional Chinese herb *Gynostemma pentaphyllum* (Thunb.) Makino, have been used to treat metabolic disorders, including lipid metabolism disorders and diabetes. Although recent studies have confirmed their beneficial effects in nonalcoholic fatty liver disease (NAFLD), the underlying therapeutic mechanism remains unclear. In this study, we explored the protective mechanism of GP against NAFLD in mice and provided new insights into the prevention and treatment of NAFLD. Male C57BL6/J mice were divided into three experimental groups: normal diet, high-fat diet (HFD), and GP groups. The mice were fed an HFD for 16 weeks to establish an NAFLD model and then treated with GP for 22 weeks. The transcriptome and proteome of the mice livers were profiled using RNA sequencing and high-resolution mass spectrometry, respectively. The results showed that GP decreased serum lipid levels, liver index, and liver fat accumulation in mice. Principal component and heatmap analyses indicated that GP significantly modulated the changes in the expression of genes associated with HFD-induced NAFLD. The 164 differentially expressed genes recovered using GP were enriched in fatty acid and steroid metabolism pathways. Further results showed that GP reduced fatty acid synthesis by downregulating the expression of *Srebf1*, *Fasn*, *Acss2*, *Acly*, *Acaca*, *Fads1*, and *Elovl6*; modulated glycerolipid metabolism by inducing the expression of *Mgll*; promoted fatty acid transportation and degradation by inducing the expression of *Slc27a1*, *Cpt1a*, and *Ehhadh*; and reduced hepatic cholesterol synthesis by downregulating the expression of *Tm7sf2*, *Ebp*, *Sc5d*, *Lss*, *Fdft1*, *Cyp51*, *Nsdhl*, *Pmvk*, *Mvd*, *Fdps*, and *Dhcr7*. The proteomic data further indicated that GP decreased the protein expression levels of ACACA, ACLY, ACSS2, TM7SF2, EBP, FDFT1, NSDHL, PMVK, MVD, FDPS, and DHCR7 and increased those of MGLL, SLC27A1, and EHHADH. In conclusion, GP can regulate the key genes involved in hepatic lipid metabolism in NAFLD mice, providing initial evidence for the mechanisms underlying the therapeutic effect of GP in NAFLD.

## Introduction

Nonalcoholic fatty liver disease (NAFLD) is the leading cause of chronic liver disease worldwide, with a global prevalence of 25% (Lazarus et al., 2022). NAFLD includes two pathologic conditions: NAFL and nonalcoholic steatohepatitis (NASH), which cover a broader spectrum of diseases, including fibrosis, cirrhosis, and hepatocellular carcinoma (EASL, EASD, EASO, 2016). NAFLD is associated with common metabolic disorders, such as obesity and type II diabetes, the prevalence of NAFLD is 55.5% in patients with type II diabetes mellitus and 80% in morbidly obese patients (Targher et al., 2021; Younossi et al., 2019). The prevalence of NAFLD is expected to continue to increase due to the increased incidence of obesity and diabetes mellitus.

Excessive lipid accumulation in the liver, which is associated with higher fat intake, directly contributes to the development and progression of NAFLD (Abenavoli et al., 2016). High-fat diet (HFD), an important factor affecting hepatic lipid metabolism, can induce NAFLD (Li et al., 2022b). Recent studies have confirmed that HFD promotes the progression of NAFLD by inducing insulin resistance and hepatic lipid accumulation, resulting in excessive accumulation of fatty acids and triglycerides, which causes severe mitochondrial dysfunction, inflammation, and apoptosis, further aggravating NAFLD development (Lian et al., 2020).

Although no drugs are currently approved for the treatment of NAFLD (Nassir, 2022), several drugs with hypoglycemic or hypolipidemic effects, such as simvastatin, metformin, and pioglitazone, have been clinically evaluated to treat NAFLD (Siddiqui et al., 2020; Feng et al., 2019; Tavakoli et al., 2022). However, these drugs have certain adverse effects. For example, long-term administration of simvastatin causes gastrointestinal toxicity and muscle injury (Muñoz et al., 2021). Therefore, investigating safe, efficient, and cost-effective anti-NAFLD drugs is clinically significant.

Gypenosides (GP) are saponin extracts isolated from *Gynostemma pentaphyllum* (Thunb.) Makino and have several pharmacological activities, including lipid metabolism regulation and atherosclerosis prevention, as well as anticancer, anti-inflammatory, antidiabetic, and anti-NAFLD effects (Li et al., 2022a; Liu et al., 2021a; Song et al., 2020; Tu et al., 2021; Weng et al., 2021; Zhu et al., 2021). GP has been reported to significantly reduce the serum levels of alanine transaminase (ALT), aspartate transaminase (AST), total cholesterol (TC), and triglycerides (TG). GP also alleviates hepatic steatosis and mitochondrial damage and downregulates the expression of inflammation-related factors, including tumor necrosis factor-alpha (TNF-α) and nuclear factor kappa B (NF-κB) in NAFLD model mice (He et al., 2015). However, due to the limited scope of previous reports, the mechanism underlying the effect of GP against NAFLD remains unclear.

Although the pharmacological effects of GP against NAFLD are well documented, the underlying molecular processes are relatively unknown. Proteins are essential for cellular processes, such as cell growth and division, organogenesis, and reproduction. Advances in transcriptomic and proteomic technologies may help identify potential targets and pathways for GP treatment of NAFLD (Lu et al., 2020). However, previous studies on the effects of GP against NAFLD have not linked the transcriptional changes to the protein level changes, which are more relevant in NAFLD.

Herein, we combined transcriptome and proteome data to explore the protective mechanism of GP in HFD-induced NAFLD mice. Transcriptomic techniques were applied to identify differentially expressed genes (DEGs) in mouse liver after GP treatment and explain the regulatory mechanisms of GP in hepatic lipid metabolism. Furthermore, the proteomic data were used to identify the correlations between the transcript and protein levels. These results might provide new insights into the mechanism of GP in treating NAFLD.

## Materials and Methods

### Materials

Male C57BL/6J mice (8 weeks old) were purchased from Huafu Kang Biological Technology Co., Ltd (Beijing, China; approval number: SCXK 2014-0004). Normal diet (ND; 23.2%, 12.1%, 64.7% of calories from proteins, fats, and carbohydrates, respectively) was purchased from Jiangsu Xietong Biomedical Engineering Co., Ltd. (Jiangsu, China). HFD (20%, 60%, and 20% of calories from proteins, fats, and carbohydrates, respectively) was purchased from Research Diets, Inc. (New Brunswick, NJ, USA) (Lu et al., 2018). GP (purity >98% assayed by UV) were purchased from Zhongxin Biotechnology Ltd. (Xian, Shanxi Province, China). TC assay kit (cat. number: A111-1-1), total triglyceride (TG) assay kit (cat. Number: A110-1-1), and LDL-cholesterol (LDL-C) assay kit (cat. Number: A113-1-1) were purchased from Nanjing Jiangcheng Bioengineering Institute (Nanjing, China). DEPC water was purchased from Zoman Biotechnology Co., Ltd. (Beijing, China).

### Animal experiments

All mice were treated in accordance with the requirements of the Animal Experiment Ethics Committee of Zunyi Medical University under the approval number [2020] 2-557. Male C57BL/6 mice (*n* = 27) were housed in groups (4–5 mice/cage) in a well-controlled specific pathogen-free room at 21–23 °C, 50–60% humidity, and a 12/12-h light/dark cycle. The animals were provided food and water *ad libitum*, and their body weight was monitored every 2 weeks. The mice were randomly divided into the following three groups (*n* = 9/group): ND, HFD, and GP groups. The mice in the ND and HFD groups were fed their respective diets for 38 weeks. The mice in the GP group were fed an HFD for 38 weeks and then gavaged with 250 mg/kg GP per day from week 17 to week 38. Whereas the mice in the ND and HFD groups were gavaged with equal volume of saline per day from week 17 to week 38 (Lu et al., 2018). At the end of the experiment, 27 mice were anesthetized with 7% chloral hydrate (0.005 mL/g) and blood was collected from their eyeballs. After euthanizing the mice *via* cervical dislocation, their livers were harvested and stored at −80 °C.

### Measurement of liver weight

After euthanizing the mice, their liver weights were measured using an Ohaus Px423zh precision balance to calculate the liver index (liver weight/body weight × 100%).

### Measurement of biochemical parameters in the serum

Serum TC, TG, and LDL-C levels of mice were measured using enzyme labeling methods according to the manufacturer’s instructions, as previously reported (Bai et al., 2021). If the serum sample volume was sufficient, three technical replicates were performed for each sample.

### Histological analysis

The liver tissues were fixed using 10% formalin for 24 h, dehydrated with low to high levels of alcohol, and then dissolved in xylene. The fixed liver tissues were embedded in paraffin (Leica EG1150; Leica, Wetzlar, Germany) and cut into 4–6-μm-thick slices using Leica RM2245 Biosystems (Wetzlar, Germany). After removing the paraffin from the slices using xylene, the slices were washed with high to low concentration alcohol and then distilled water. Finally, the slices were stained using hematoxylin and eosin (H&E) for examination under an Olympus BX43 microscope (Tokyo, Japan).

### RNA preparation and sequencing

The livers of five mice in each group were randomly selected for RNA-seq. Approximately 20 mg of the liver tissue was homogenized in 1 mL of Trizol on ice, incubated for 5 min, and 200 μL of chloroform was added. After centrifugation (12,000 *g*) at a low temperature (4 °C), the RNA was precipitated with 500 μL of isopropanol. The precipitate was washed with 75% ethanol and then dissolved in 30 μL of DEPC water. RNA integrity was evaluated using an Agilent 2100 Bioanalyzer using the specific eligibility criteria (RNA integrity number ≥ 6.5, 28S:18S ≥ 1). RNA sequencing was performed on an Illumina Hiseq 2000 platform. The statistical power of this experimental design, as calculated in RNASeqPower, is 0.99. RNA-seq data generated in this study have been submitted to the NCBI BioProject database (https://www.ncbi.nlm.nih.gov/sra/) under the accession number PRJNA885754.

### Protein extraction

The liver samples were ground into powder under liquid nitrogen and transferred to a 5-mL centrifuge tube. Then, four volumes of lysis buffer (8 M urea, 1% protease inhibitor cocktail) were added to this powder. After sonication, the protein extracts were centrifuged at 12,000×*g* for 10 min. Finally, the supernatant was collected and the protein concentration was determined using the BCA kit according to the manufacturer’s instructions. The protein was purified *via* sodium dodecyl sulfate-polyacrylamide gel electrophoresis (SDS-PAGE). The trapped proteins were digested overnight using trypsin. The eluted peptides were desalted and centrifuged at 14,000×*g* for 15 min, and the supernatant was collected for LC–MS/MS analysis. Data were collected as described previously (Wang et al., 2021). The mass spectrometry proteomics data have been deposited in the ProteomeXchange Consortium (http://proteomecentral.proteomexchange.org) *via* the iProX partner repository (Chen et al., 2022; Ma et al., 2019) with the dataset identifier PXD038829.

### Data processes and mining

We generated a list of 1,310 genes involved in NAFLD using the nash-profiler database (http://www.nash-profiler.com). The R program was used to visualize the expression levels of all investigated NAFLD genes and profile their global changes (R Core Team, 2023). Principal component analysis (PCA) was used to statistically evaluate the gene expression using individual mice as samples. Each gene expression value of a single sample was used as a variable to construct a data matrix, which was processed by the PCA function of mixOmics in the R language open-source toolkit. Gene Ontology (GO) analysis was performed using the enrichGO function in the clusterProfiler package. Venn analysis was performed in the R project VennDiagram package. Differences between the two groups were calculated using t-test in R, whereas those among multiple groups were calculated using one-way ANOVA in R. *P* < 0.05 represented statistical significance, and all data are expressed as mean ± standard error (SEM). Outliers with values >3 standard deviations (SD) were used as the exclusion criteria.

## Results

### Effects of GP on serum lipids and liver pathology in mice

**Figure 1:**
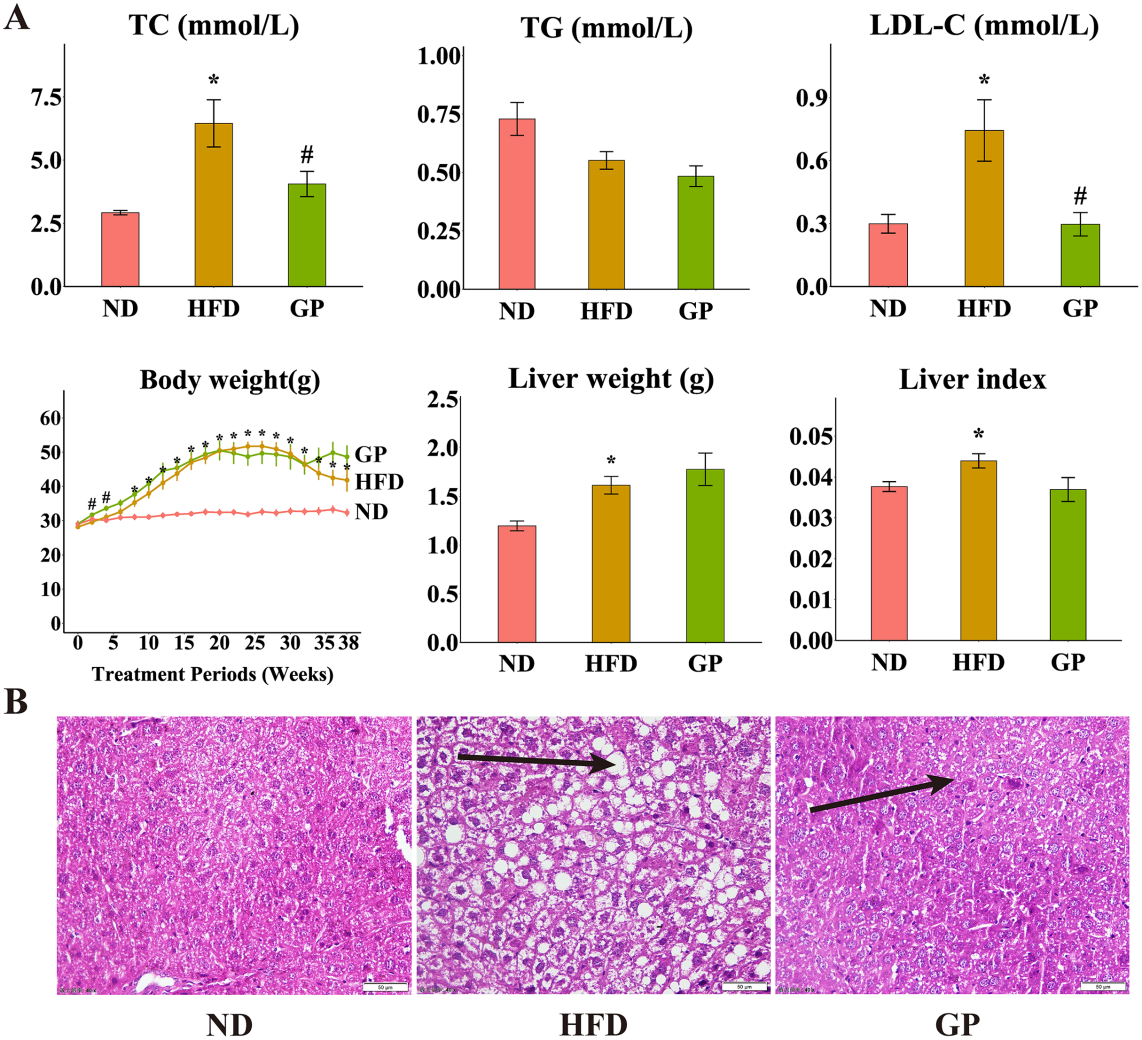
Effect of GP on mice. (A) The impact of GP on TC, TG, LDL-C, body weight, liver weight and liver index. (B) Liver histological examination with H&E (200×). Each bar represents the mean ± the SEM of the values obtained from nine animals. *p < 0.05 *vs*. ND group, ^#^*p* < 0.05 *vs*. HFD group.

Compared with the ND group, the HFD group showed significantly increased levels of TC (120.6%, *P* < 0.05), LDL-C (148.4%, *P* < 0.05), liver weight (34.9%, *P* < 0.05), and liver index (11.7%, *P* < 0.05) but decreased level of TG (32.2%, *P* = 0.17). Compared with the HFD group, the GP group showed reduced TC (4.06 ± 0.50 *vs*. 6.45 ± 0.93, *P* < 0.05) and LDL-C (0.30 ± 0.06 *vs*. 0.74 ± 0.15, *P* < 0.05) levels and liver index (0.037 ± 0.003 *vs*. 0.044 ± 0.003, *P* = 0.056), without any significant changes in TG level (0.48 ± 0.04 *vs*. 0.55 ± 0.04, *P* = 0.17) and liver weight (1.78 ± 0.17 *vs*. 1.61 ± 0.09, *P* = 0.40). Compared with the ND group, the HFD group showed significantly increased body weight after week 8 (*P* < 0.05). Compared with the HFD group, the GP group showed significantly increased body weight in the first 4 weeks (*P* < 0.05), which apparently increased after 32 weeks, but not significantly (Fig. 1A). H&E staining showed that GP significantly reversed HFD-induced liver fat accumulation in mice (Fig. 1B).

### GP modulated changes in the expression of genes involved in HFD-induced NAFLD

**Figure 2:**
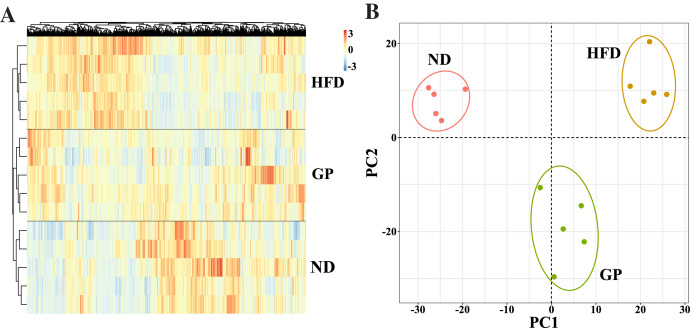
Comparison of transcriptome profiling. (A) Heatmap analysis of three groups. (B) PCA analysis of three groups.

We performed RNA-seq using the liver tissues of GP-treated NAFLD mice to explore the protective mechanism of GP against NAFLD. A hierarchical clustering heat map showed that the ND, HFD, and GP groups clustered separately, with changes in gene expression in response to HFD being opposite to those in response to GP treatment (Fig. 2A). This was further confirmed using PCA, where a trend of spatial separation was observed between the HFD and ND groups and between the GP and HFD groups. This indicated that GP reversed the HFD-induced transcriptional changes in the direction of PC1, suggesting that GP significantly ameliorated the HFD-induced changes in NAFLD-related gene expression (Fig. 2B).

### Identification of DEGs across diseases and therapies

**Figure 3:**
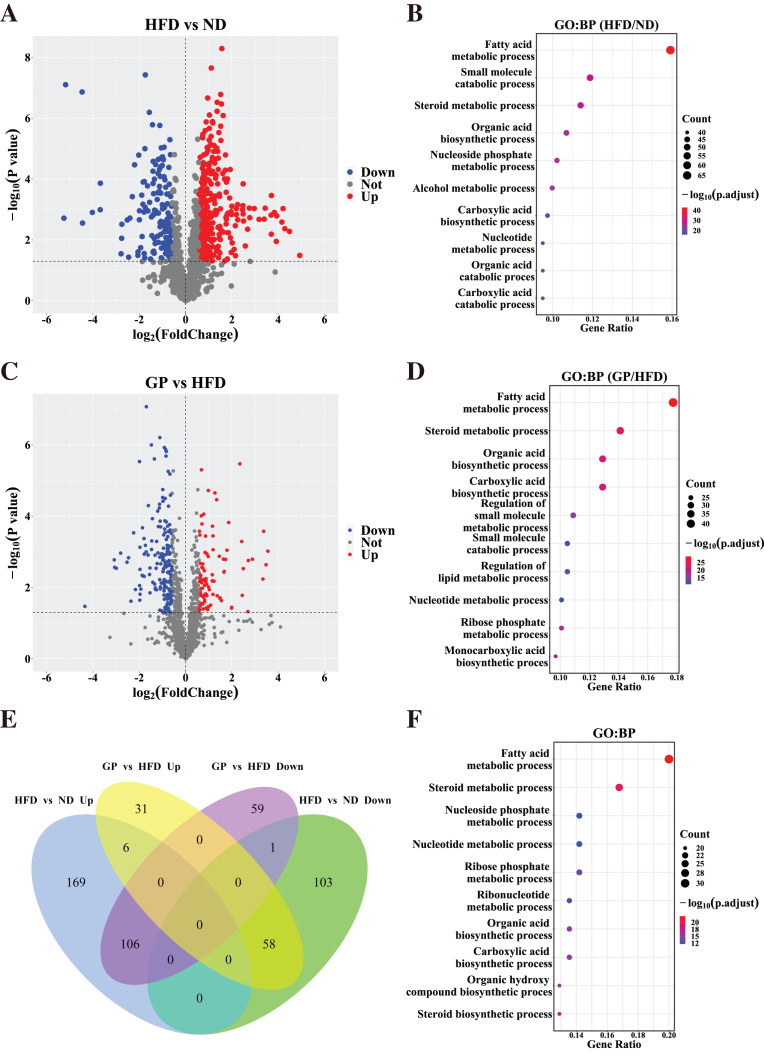
Identification of differentially expressed genes. (A) The volcano plots for the distribution of DEGs between HFD and ND group. (B) GO pathway enrichment analysis based on DEGs between HFD and ND group. The bubble color is related to the *p* value, and the bubble size is related to the number of genes enriched in this pathway. (C) The volcano plots for the distribution of DEGs between GP and HFD group. (D) GO pathway enrichment analysis based on DEGs between GP and HFD group. (E) The Venn diagram shows an overlap among the HFD/ND and GP/HFD genes. We obtained 485 (278 + 207) intersection targets which were changed by HFD and then returned by GP. (F) GO pathway enrichment analysis based on shared genes that are back-regulated by GP. Abbreviation: Up, significant up-regulated; Down, significant down-regulated; Not, no changed; Count, the number of the differential expressed genes enriched in each term.

Based on the gene expression data, we generated a volcano plot showing HFD-mediated changes in these genes (Fig. 3A). Compared with the ND group, 443 DEGs (Fold change (FC) > 1.5, *P* < 0.05), including 281 upregulated genes and 162 downregulated genes, were observed in the HFD group. GO enrichment analysis showed that the DEGs affected by HFD were mainly involved in fatty acid and steroid metabolism and small molecule catabolism (Fig. 3B).

To understand how GP treatment affects NAFLD, we compared the transcriptome profiles of the HFD and GP groups. Compared with the HFD group, 261 DEGs, including 95 upregulated genes and 166 downregulated genes, were observed in the GP group (Fig. 3C). GO enrichment analysis showed that the DEGs were mainly involved in fatty acid and steroid metabolism and organic acid biosynthesis (Fig. 3D).

The Venn diagram in Fig. 3E shows the union and shared DEGs. A total of 171 overlapping genes were found, 164 of which were recovered *via* GP treatment. GO enrichment analysis showed that the abovementioned 164 DEGs were mainly involved in fatty acid, steroid, and nucleoside phosphate metabolism pathways (Fig. 3F). These results indicate that GP improves NAFLD mainly by regulating lipid metabolism.

### GP regulated hepatic fatty acid metabolism

**Figure 4:**
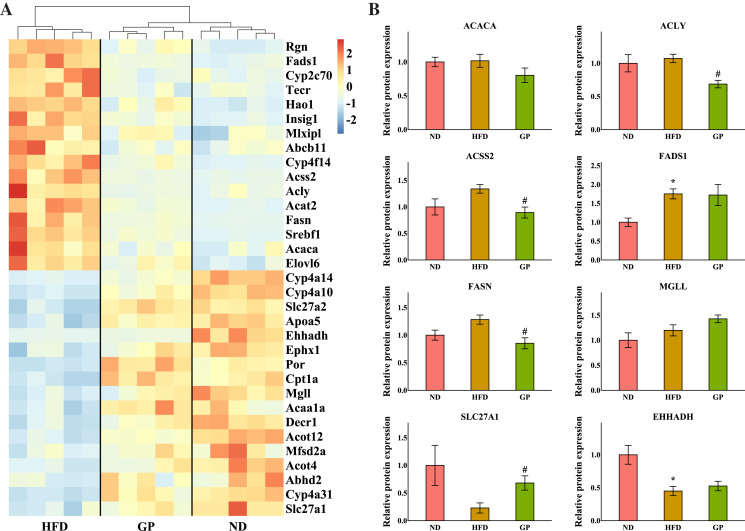
Effects of GP on fatty acid metabolic process. (A) The expression profile of DEGs implicated in the metabolism of fatty acid was shown as heatmap. The color scheme (red to blue) represents the up to down of the gene expression. (B) The protein level of key enzymes. An asterisk (*) indicates *p* < 0.05 *vs.* ND group, # indicates *p* < 0.05 *vs.* HFD group.

The heatmap in Fig. 4A shows the expression profile of DEGs enriched for fatty acid metabolism based on GO analysis. Sterol regulatory element-binding protein 1 (*Srebf1*), fatty acid synthase (*Fasn*), acetyl-CoA synthetase 2 (*Acss2*), ATP-citrate lyase (*Acly*), acetyl-CoA carboxylases alpha (*Acaca*), fatty acid desaturase 1 (*Fads1*), and ELOVL fatty acid elongase 6 (*Elovl6*), which are the key genes for fatty acid synthesis, were all downregulated by GP (Srebf1: 0.26-fold; Fasn: 0.22-fold; Acss2: 0.29-fold; Acly: 0.26-fold; Acaca: 0.61-fold; Fads1: 0.50-fold; and Elovl6: 0.61-fold decrease, all *P* < 0.05). Contrastingly, several genes responsible for TG and fatty acid metabolism and fatty acid transportation, including carnitine palmitoyltransferase 1A (*Cpt1a*), enoyl-CoA hydratase and 3-hydroxyacyl CoA dehydrogenase (*Ehhadh*), monoglyceride lipase (*Mgll*), and solute carrier family 27 member 1 (*Slc27a1*), were significantly increased after GP treatment (Cpt1a: 2.41-fold; Ehhadh: 2.28-fold; Mgll: 1.79-fold; and Slc27a1: 1.85-fold increase, all *P* < 0.05). To further explore whether RNA-seq data were consistent with the protein levels, we selected the most relevant genes in fatty acid metabolism to match the proteomic data (Fig. 4B). As represented, the levels of ACACA, ACLY, ACSS2, and FASN were lower in the GP group than in the HFD group (ACACA: 0.79-fold, *P* = 0.18; ACLY: 0.64-fold, *P* < 0.05; ACSS2: 0.67-fold, *P* < 0.05; and FASN: 0.66-fold decrease, *P* < 0.05). Meanwhile, the MGLL, SLC27A1, and EHHADH levels were higher in the GP group compared with HFD group(MGLL: 1.2-fold, *P* = 0.12; SLC27A1: 2.96-fold, *P* < 0.05; and EHHADH: 1.17-fold increase, *P* = 0.47).

### GP regulated hepatic steroid metabolism

**Figure 5:**
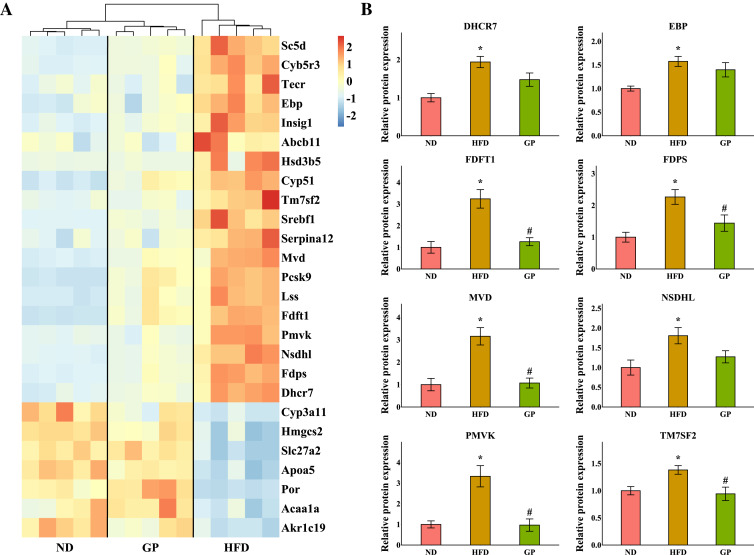
Effects of GP on steroid metabolic process. (A) The expression profile of DEGs implicated in the metabolism of steroid was shown as heatmap. The color scheme (red to blue) represents the up to down of the gene expression. (B) The protein level of key enzymes. An asterisk (*) indicates *p* < 0.05 *vs.* ND group, # indicates *p* < 0.05 *vs.* HFD group.

The heatmap shows the expression profile of DEGs enriched for steroid metabolism based on GO analysis (Fig. 5A). Essential genes involved in steroid metabolism, including transmembrane 7 superfamily member 2 (*Tm7sf2*), EBP cholestenol delta-isomerase (*Ebp*), sterol-C5-desaturase (*Sc5d*), lanosterol synthase (*Lss*), farnesyl diphosphate farnesyltransferase 1 (*Fdft1*), cytochrome P450, family 51 (*Cyp51*), NAD(P) dependent steroid dehydrogenase-like (*Nsdhl*), phosphomevalonate kinase (*Pmvk*), mevalonate diphosphate decarboxylase (*Mvd*), farnesyl diphosphate synthase (*Fdps*), and 7-dehydrocholesterol reductase (*Dhcr7*), were all significantly downregulated by GP (Tm7sf2: 0.42-fold; Ebp: 0.6-fold; Sc5d: 0.43-fold; Lss: 0.54-fold; Fdft1: 0.64-fold; Cyp51: 0.54-fold; Nsdhl: 0.28-fold; Pmvk: 0.36-fold; Mvd: 0.37-fold; Fdps: 0.29-fold; and Dhcr7: 0.36-fold decrease, all *P* < 0.05). Proteomic data also indicated that the expression levels of TM7SF2, EBP, FDFT1, NSDHL, PMVK, MVD, FDPS, and DHCR7 were downregulated by GP (TM7SF2: 0.68-fold, *P* < 0.05; EBP: 0.89-fold, *P* = 0.37; FDFT1: 0.38-fold, *P* < 0.05; NSDHL: 0.70-fold, *P* = 0.07; PMVK: 0.29-fold, *P* < 0.05; MVD: 0.34-fold, *P* < 0.05; FDPS: 0.64-fold, *P* < 0.05; and DHCR7: 0.76-fold, *P* = 0.08) (Fig. 5B).

## Discussion

The increasing incidence of NAFLD is one of the most common risk factors for advanced liver disease and related metabolic complications and has become a significant burden on the economy and healthcare system. Unfortunately, no drugs are currently approved for the treatment of NAFLD (Nassir, 2022). In the present study, we observed that GP were potentially effective in treating NAFLD as it significantly reduced the serum levels of ALT, AST, TC, and TG. GP also improved hepatic steatosis and insulin resistance and reduced inflammatory liver injury in HFD-induced NAFLD mice. These results were consistent with those of a previous study (Li et al., 2022a). In addition, we applied transcriptome analysis to further assess the specific effects of GP against NAFLD. Heatmap visualization and PCA showed that GP improved the abnormal transcriptome profiles in HFD-fed mice. GO analysis suggested that the DEGs restored by GP were significantly enriched in fatty acid and steroid metabolism (Figs. 3E, 3F). This finding suggested that GP ameliorates NAFLD in mice by broadly regulating the expression of genes involved in lipid metabolism.

**Figure 6:**
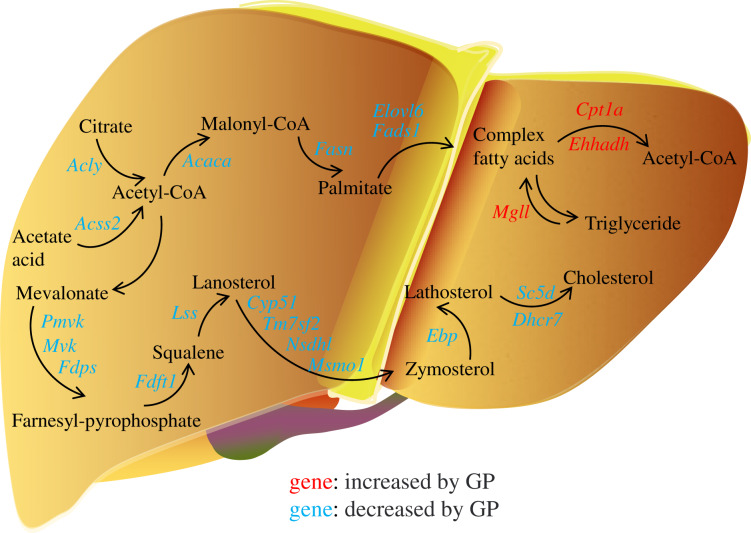
Regulation of gypenosides on genes related to fatty acid and cholesterol metabolic pathways in mice liver. The red font represents that the expression of this gene is significantly decreased under the influence of high fat, and significantly increased after the treatment of gypenosides. The blue font represents that the expression of this gene is significantly increased under the influence of high fat, and significantly decreased after the treatment of gypenosides.

In the pathogenesis of NAFLD, disruption of the balance between fatty acid biosynthesis and degradation is the main cause of dysregulated lipid metabolism. Glucose derived from the diet produces citrate in the mitochondria, which is transported to the cytosol and catalyzed by ACLY to release acetyl-CoA. ACSS2 can also convert acetate acid to acetyl-CoA (Zhao et al., 2020), which is then converted to malonyl-CoA by ACACA. FASN, the key rate-limiting enzyme in *de novo* lipogenesis, converts malonyl-CoA into palmitate. FADS1 and ELOVL6 catalyze the elongation and desaturation of palmitate to produce complex fatty acids, including stearic, oleic, and palmitoleic acids (Song, Xiaoli & Yang, 2018) (Fig. 6). SREBP1, encoded by *Srebf1*, acts as the master regulator of fatty acid biogenesis and sterol metabolism by transcriptionally regulating the expression of genes, including *Acly*, *Acaca*, and *Fasn* (Zhou et al., 2022). When there is excess energy in the body, the newly synthesized fatty acids in the liver are mainly esterified to triglycerides for storage, promoting the development of fatty liver disease. Recent studies have shown that inhibiting SREBP1, ACLY, ACSS2, ACACA, and FASN can improve fatty liver disease (Knebel et al., 2018; Morrow et al., 2022; Huang et al., 2018; Chen et al., 2019; Loomba et al., 2021). In the present study, we found that the expression levels of the genes *Srebf1*, *Acly*, *Acss2*, *Acaca*, *Fasn*, *Elovl6*, and *Fads1* and proteins ACLY, ACSS2, ACACA, FASN decreased after GP treatment in HFD-fed mice (Fig. 4). These findings suggest that GP exert their anti-NAFLD effects by inhibiting the synthesis and accelerating the metabolism of fatty acids.

MGLL is an important fatty acid metabolism enzyme that converts triglycerides to free fatty acids (Zhang et al., 2021). CPT1A is responsible for transporting free fatty acids from the cytoplasm to the mitochondria, which enables fatty acid oxidation (Hao et al., 2021). EHHADH is a crucial participant in the classical peroxisomal fatty acid β-oxidation pathway, which is the primary lipid catabolism pathway (Hu et al., 2021) (Fig. 6). SLC27A1, a transporter for cellular fatty acid uptake, can enhance fatty acid oxidation and inhibit muscle fat accumulation (Huang, Zhu & Shi, 2021). Recent studies have shown that increased expression of EHHADH and CPT1A is beneficial for the treatment of NAFLD (Huang et al., 2022). In the present study, the expressions of the genes *Mgll*, *Cpt1a*, *Ehhadh*, and *Slc27a1* and the protein SLC27A1increased after GP treatment in HFD-fed mice (Fig. 4). These results further confirmed that GP could accelerate fatty acid metabolism.

In addition, the accumulation of liver cholesterol contributes to the pathogenesis of NAFLD. The steroid metabolism process in the liver mainly includes the synthesis and metabolism of cholesterol and the metabolism of its downstream product, steroid hormones. Excess hepatic cholesterol preferentially accumulates in hepatocyte lipid droplets, which may crystallize and contribute to the development of NASH (Bashiri et al., 2016; Ioannou et al., 2019). Mevalonate is catalyzed by PMVK, MVD, and FDPS to generate farnesyl pyrophosphate. FDFT1 mediates squalene production, which is catalyzed by LSS to generate lanosterol. Then, CYP51, TM7SF2, NSDHL, and MSMO1 catalyze the formation of zymosterol, which is further catalyzed by EBP to generate lathosterol. Finally, cholesterol is synthesized *via* the catalytic action of SC5D and DHCR7 (Luo, Yang & Song, 2020; Benson et al., 2017; Ershov et al., 2021; Wei et al., 2021) (Fig. 6). Recent studies have shown that reducing the expression of FDFT1, SQLE, LSS, CYP51, TM7SF2, and EBP is beneficial in the treatment of NAFLD (Dong et al., 2020; Liu et al., 2021b; Kanoni et al., 2021; Li et al., 2020; Huang et al., 2021; Chang et al., 2021). We showed that the expression of key genes involved in cholesterol synthesis was significantly increased in the HFD group but decreased in the GP group. Proteomic data also demonstrated that GP decreased the expression levels of DHCR7, EBP, FDFT1, FDPS, MVD, NSDHL, PMVK, and TM7SF2 (Fig. 5). These results suggested that GP could inhibit cholesterol synthesis.

## Conclusions

In conclusion, our findings suggest that GP can be used to treat NAFLD by simultaneously inhibiting the synthesis of fatty acids and cholesterol and accelerating the metabolism of fatty acids.

## Supplemental Information

10.7717/peerj.15225/supp-1 Supplemental Information 1 Cholesterol.

10.7717/peerj.15225/supp-2 Supplemental Information 2 Triglyceride.

10.7717/peerj.15225/supp-3 Supplemental Information 3 LDL-C.

10.7717/peerj.15225/supp-4 Supplemental Information 4 Proteome expression.

10.7717/peerj.15225/supp-5 Supplemental Information 5 Body weight.

10.7717/peerj.15225/supp-6 Supplemental Information 6 Codes.

10.7717/peerj.15225/supp-7 Supplemental Information 7 RNA expression（FPKM）.

10.7717/peerj.15225/supp-8 Supplemental Information 8 Liver weight and body weight.

10.7717/peerj.15225/supp-9 Supplemental Information 9 Proteome sequencing.

10.7717/peerj.15225/supp-10 Supplemental Information 10 Author Checklist.

## Additional Information and Declarations

### DNA Deposition

The following information was supplied regarding the deposition of DNA sequences: The RNA sequencing is available at NCBI: PRJNA885754. The mass spectrometry proteomics data are available at the ProteomeXchange Consortium *via* the iProX partner repository: PXD038829.

### Data Availability

The following information was supplied regarding data availability: The raw measurements and code are available in the Supplemental Files.
